# Characteristics of progressive temporal visual field defects in patients with myopia

**DOI:** 10.1038/s41598-021-88832-1

**Published:** 2021-04-30

**Authors:** Jiyun Lee, Chan Kee Park, Kyoung In Jung

**Affiliations:** Department of Ophthalmology, Seoul St. Mary’s Hospital, College of Medicine, The Catholic University of Korea, 222, Banpo-daero, Seocho-gu, 06591 Seoul, Republic of Korea

**Keywords:** Glaucoma, Optic nerve diseases, Refractive errors

## Abstract

Temporal visual field damage (VFD) is the common type of non-glaucomatous VF defects found in eyes with myopia. However, little is known about the factors associated with its progression. We investigated the characteristic of myopic eyes with progressive temporal VF defects. This retrospective, observational study included a total of 116 eyes: 39 eyes with temporal VFDs and an axial length greater than 24.5 mm, 77 eyes with typical glaucomatous VFDs who were followed up more than 5 years. VF progression was evaluated with Trend-based global progression analysis. In the temporal VFD group, the greater tilt ratios, the higher prevalence of β-zone peripapillary atrophy (β-PPA), the substantial increase in β-PPA were found, compared to the typical glaucomatous VFD groups (all *P*-values ≤ 0.001). The temporal VFD group had the slower progression than the typical glaucomatous VFD group on trend-based GPA (*P* = 0.047). In the multivariate linear regression analysis, the change of β-PPA area over years was related to temporal VFD progression (B, − 0.000088, *P* = 0.003). In conclusion, myopic eyes with the temporal VFD, which come with growing β-PPA area, should be monitored with extra caution.

## Introduction

Myopia has become increasingly prevalent worldwide, especially in Asia^1^. Myopia could be a critical public health problem because it is associated with a risk of visual impairment^2^. In addition, myopia has been regarded as an independent risk factor for the development of glaucoma, and its high prevalence among glaucoma patients has been supported by epidemiological reports^3,4^.

Myopic eyes undergo the elongation of the posterior segment, and this elongation would result in structural changes to the optic nerve head (ONH) including tilt, torsion, and peripapillary atrophy (PPA)^5–7^. Considering the fact that diagnosis of glaucoma is based on characteristic ONH changes such as retinal nerve fiber layer (RNFL) loss with cupping, these bizarre degenerations around ONH may interrupt the accurate diagnosis of glaucoma based on structural changes.

In addition to ONH changes, visual field defect (VFD) is another essential requirement for the diagnosis of glaucoma. Therefore, when myopic patients present with stereotypical glaucomatous VFDs, the diagnosis is relatively easy and treatment can be initiated without hesitation. However, when it comes to atypical VFDs accompanied by structural myopic changes in the ONH, it is hard to decide whether the patient needs treatment.

Regarding VFDs in myopic eyes, several reports found that temporal VFDs, including enlarged blind spots, were the most frequently observed VFD in myopic patients^8,9^. In true glaucoma, however, the temporal VFD is not regarded as a typical type of the VFDs.

One study reported that some high myopic eyes without definite glaucoma showed a significant progression of VFDs^9^. Another study revealed that a gradual decrease in peripapillary RNFL thickness was observed, especially in older patients with high myopia^10^. Given those findings, there is a possibility that myopia-related VFD can progress, even though that is not a stereotypical glaucomatous VFD.

Little is known about the factors associated with the progression of VF damage in myopic patients with non-glaucomatous VFDs including temporal VFD. Understanding the clinical characteristics associated with the progression of VFD in these patients might help to provide the proper approach and better management for these populations.

In this study, we investigated the factors related to temporal VFDs and its progression in myopic patients, focusing on the temporal VFDs in myopic patients as non-classical glaucomatous VFDs. In addition, the characteristics of myopic patients with temporal VFDs were compared to those of patients with typical early glaucomatous VFDs.

## Results

Among initially recruited 243 eyes, 127 eyes were excluded: 55 eyes due to less than three VF tests; 23 eyes with far advanced VFD; 33 eyes with VFD involving both periphery and center of VF test points; 12 eyes invading both the edge and periphery of the field; and 4 eyes with neurological disease.

A total of 116 eyes of 116 patients, with the mean follow-up 80.34 ± 18.56 months, were finally participated in this study, and according to VFD location, the numbers of patients are following: 39 myopic eyes with the temporal VFD and 79 eyes with typical glaucomatous VFD. Typical glaucomatous VFD group included 37 eyes with the peripheral VFD and 40 eyes with the paracentral VFD.

The patient demographics are shown in Table 1. Patients in the temporal VFD group had the significantly higher prevalence of hypertension (*P* = 0.025). As for glaucoma medication, although more than two third of the temporal VFD group was on glaucoma medication, the percentage of the patient and the number of the eyedrops were substantially lower compared to typical glaucomatous group. Regarding axial length, the temporal VFD group had the longer eyes(*P* < 0.001).

**Table 1:** Patients demographics (Total 116 eyes).

		Temporal VFD (N, 39)	Typical glaucomatous VFD (N, 77)	*P* value
Age, mean (SD), year		52.49 (13.66)	56.12 (12.65)	0.158^a^
Female, No. (%)		30 (76.9)	47 (61.0)	0.087^b^
Laterality, No. (%)	OD	21 (53.8)	40 (51.9)	0.847^b^
	OS	18 (46.2)	37 (48.1)	
HTN (No. (%))		1 (2.6)	13 (16.9)	**0.025**^**b**^
DM (No. (%))		2 (5.1)	4 (5.2)	0.988^b^
Aspirin (No. (%))		0 (0)	4 (5.2)	0.147^b^
Migraine (No. (%))		4 (10.3)	7 (9.1)	0.840^b^
Cold hands/ feet (No. (%))		4 (10.3)	310(13.0)	0.670^b^
Baseline IOP, mean (SD), mmHg		16.08 (3.32)	15.44 (3.19)	0.320^a^
Final IOP, mean (SD), mmHg		15.00 (2.86)	14.25 (3.14)	0.211^a^
Number of patients with medication (No. (%))		28 (71.8)	77 (100)	** < 0.001**^**b**^
Number of medications, mean (SD), n		1.03 (0.81)	1.75 (0.79)	** < 0.001**^**a**^
CCT, mean (SD), μm		536.34 (55.96)	536.74 (36.17)	0.969^a^
Axial length, mean (SD), mm		27.74 (2.29)	24.70 (1.62)	** < 0.001**^**a**^
Number of VF test, mean (SD), n		6.59 (1.39)	6.10 (1.10)	0.052^a^
Total follow-up period, mean (SD), month		86.43 (14.49)	81.90 (20.20)	0.072^a^
Visual field test	MD, mean (SD), dB	− 3.23 (2.84)	− 2.98 (1.63)	0.618^a^
	PSD, mean (SD), dB	4.83 (2.16)	4.94 (2.37)	0.807^a^

*VFD* visual field defect, *HTN* hypertension, *DM* diabetes mellitus, *IOP* intraocular pressure, *n* number, *CCT* central corneal thickness, *VF* visual field, *MD* mean deviation, *PSD* pattern standard deviation, *dB* decibel.

Mean values are presented with standard deviations ^a^Student’s t-test after Propensity score match, ^b^chi-squared test.

Bold font indicates significant *P* values (*P* < 0.05).

**Figure 1:**
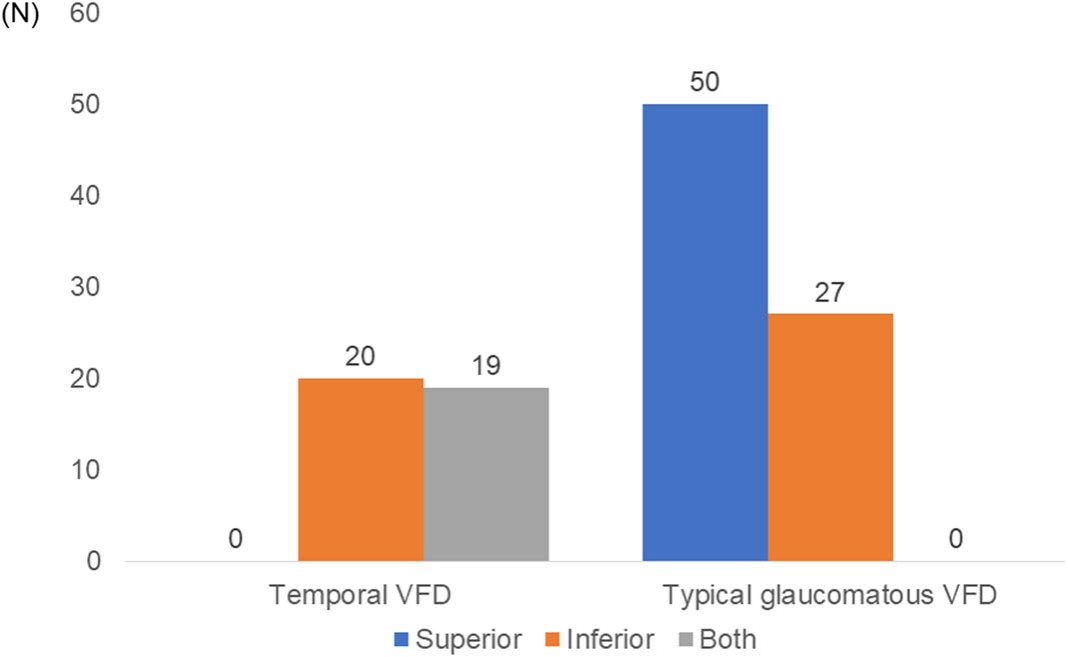
Location of Visual Field Defect in All eyes (N, 74). Distributions of visual field defect in each hemisphere are shown. (chi-squared test, *P* < 0.001). *VFD* visual field defect.

Distribution of each VFD group in hemispheres was different. For the temporal VFD groups, VF loss was prominent in inferior hemisphere or in both hemispheres. Unlike the temporal VFD group, the typical glaucomatous VFD group saw a noticeable VF loss in superior hemisphere, and the difference between the two groups was significant. (*P* < 0.001) (Fig. 1).

The β-PPA measurements showed excellent reproducibility for the initial and final β-PPA areas (ICC = 0.874–0.996, ICC = 0.907–0.981, respectively), final disc area (0.871–0.988) (Table 2). The reproducibility for initial disc area was moderate to excellent (ICC = 0.710–0.778).

**Table 2:** Reliability of β-zone PPA parameters.

Variables		Temporal VFD (N, 39)		Typical glaucomatous VFD (N, 77)	
		ICC	95% CI	ICC	95% CI
β-zone PPA	Area of Initial β-zone PPA, mean (SD), pixel	0.996	0.992–0.998	0.874	0.681–0.950
	Area of Initial disc, mean (SD), pixel	0.778	0.488–0.904	0.710	0.267–0.885
	Area of Final β-zone PPA, mean (SD), pixel	0.907	0.786–0.960	0.981	0.954–0.992
	Area of Final disc, mean (SD), pixel	0.871	0.702–0.944	0.988	0.970–0.995

*VFD* visual field defect, *ICC* intraclass correlation coefficient, *PPA* peripapillary atrophy.

Reproducibility of β-zone PPA parameters: excellent for areas of initial and final β-zone PPA of both groups, final disc areas of both groups, and initial disc area of temporal VFD group; moderate for initial disc area of typical glaucomatous VFD group.

The parameters related to optic discs and OCT were compared between two groups (Tables 3 and 4). The prevalence of the optic disc tilt and the degree of the tilt ratio were significantly greater in the temporal VFD group, compared to the other group (All *P*-values ≤ 0.001). All parameters related to β-PPA showed significantly greater values in the temporal VFD group than in the typical glaucomatous VFD group (All *P*-values < 0.001). As to OCT parameters (Table 4), the typical glaucomatous VFD group saw the substantially lower values in average, nasal, and temporal RNFL thicknesses, and average, inferonasal, inferior, and inferotemporal GCIPL thicknesses compared to the temporal VFD group.

**Table 3:** Comparison of optic disc and posterior pole profiles.

Variables		Temporal VFD (N, 39)	Typical glaucomatous VFD (N, 77)	*P* value
Manually measured cup to disc ratio, mean (SD)		0.67 (0.21)	0.70 (0.17)	0.349^a^
Presence of Disc hemorrhage, (No. (%))		3 (7.7)	14 (18.2)	0.131^b^
Optic disc tilt	Presence of Tilted disc (No. (%))	28 (71.8)	17 (22.1)	** < 0.001**^**b**^
	Tilt ratio, mean (SD)	1.42 (0.23)	1.21 (0.30)	** < 0.001**^**a**^
Optic disc rotation	Rotation angle, mean (SD), °	5.04 (19.42)	− 1.00 (20.87)	0.136^a^
	Significant rotation (No. (%))	16 (41.0)	41 (54.7)	0.167^b^
	Inferior rotation (No. (%))	29 (74.4)	38 (50.7)	**0.015**^**b**^
β-zone PPA	Presence (No. (%))	34 (87.2)	39 (50.6)	** < 0.001**^**b**^^27^
	Area of Initial β-zone PPA, mean (SD), pixel	62,451.3 (52,461.7)	13,369.8 (7316.6)	** < 0.001**^**a**^
	Change of β-zone PPA over years, mean (SD), pixel/year	3285.5 (3585.9)	237.4 (782.8)	** < 0.001**^**a**^

*VFD* visual field defect, *PPA* peripapillary atrophy.

^a^Student’s t-test after Propensity score match, ^b^chi-squared test.

Bold font indicates significant *P* values (*P* < 0.05).

**Table 4:** Comparison of optical coherence tomography parameters.

Variable		Temporal VFD (N, 39)	Typical glaucomatous VFD (N, 77)	*P* value
Optic disc parameters	Rim area, mean (SD)	1.47 (0.78)	0.90 (0.20)	** < 0.001**
	Disc area, mean (SD)	2.76 (1.43)	2.03 (0.59)	**0.005**
	Average cup to disc ratio, mean (SD)	0.82 (0.23)	0.75 (0.14)	0.072
	Vertical cup to disc ratio, mean (SD)	0.75 (0.28)	0.74 (0.14)	0.812
	Cup volume, mean (SD)	0.30 (0.25)	0.48 (0.30)	**0.004**
Parapapillary RNFL thickness (μm)	Average, mean (SD)	86.97 (16.14)	77.88 (9.50)	**0.003**
	Superior, mean (SD)	92.70 (24.49)	95.86 (20.10)	0.484
	Nasal, mean (SD)	74.58 (24.06)	63.58 (9.54)	**0.010**
	Inferior, mean (SD)	96.91 (24.93)	87.98 (16.76)	0.056
	Temporal, mean (SD)	83.96 (23.56)	64.23 (14.96)	** < 0.001**
GCIPL thickness (μm)	Average, mean (SD)	78.13 (9.64)	71.01 (7.08)	** < 0.001**
	Minimum, mean (SD)	64.89 (19.04)	58.87 (10.77)	0.105
	Superior, mean (SD)	73.27 (14.63)	73.47 (9.90)	0.945
	Superonasal, mean (SD)	81.38 (17.05)	78.52 (7.83)	0.373
	Inferonasal, mean (SD)	80.17 (13.42)	73.77 (8.34)	**0.005**
	Inferior, mean (SD)	73.01 (12.39)	64.64 (10.65)	** < 0.001**
	Inferotemporal, mean (SD)	79.81 (14.39)	63.54 (10.93)	** < 0.001**
	Superotemporal, mean (SD)	80.85 (15.31)	71.26 (10.00)	0.309

Values obtained from optical coherence tomography was corrected with the Littmann’s formula.

*VFD* visual field defect, *RNFL* retinal nerve fiber layer, *GCIPL* ganglion cell inner plexiform layer.

Mean values are presented with standard deviations.

Student’s t-test after Propensity score match.

Bold font indicates significant *P* values (*P* < 0.05).

**Figure 2:**
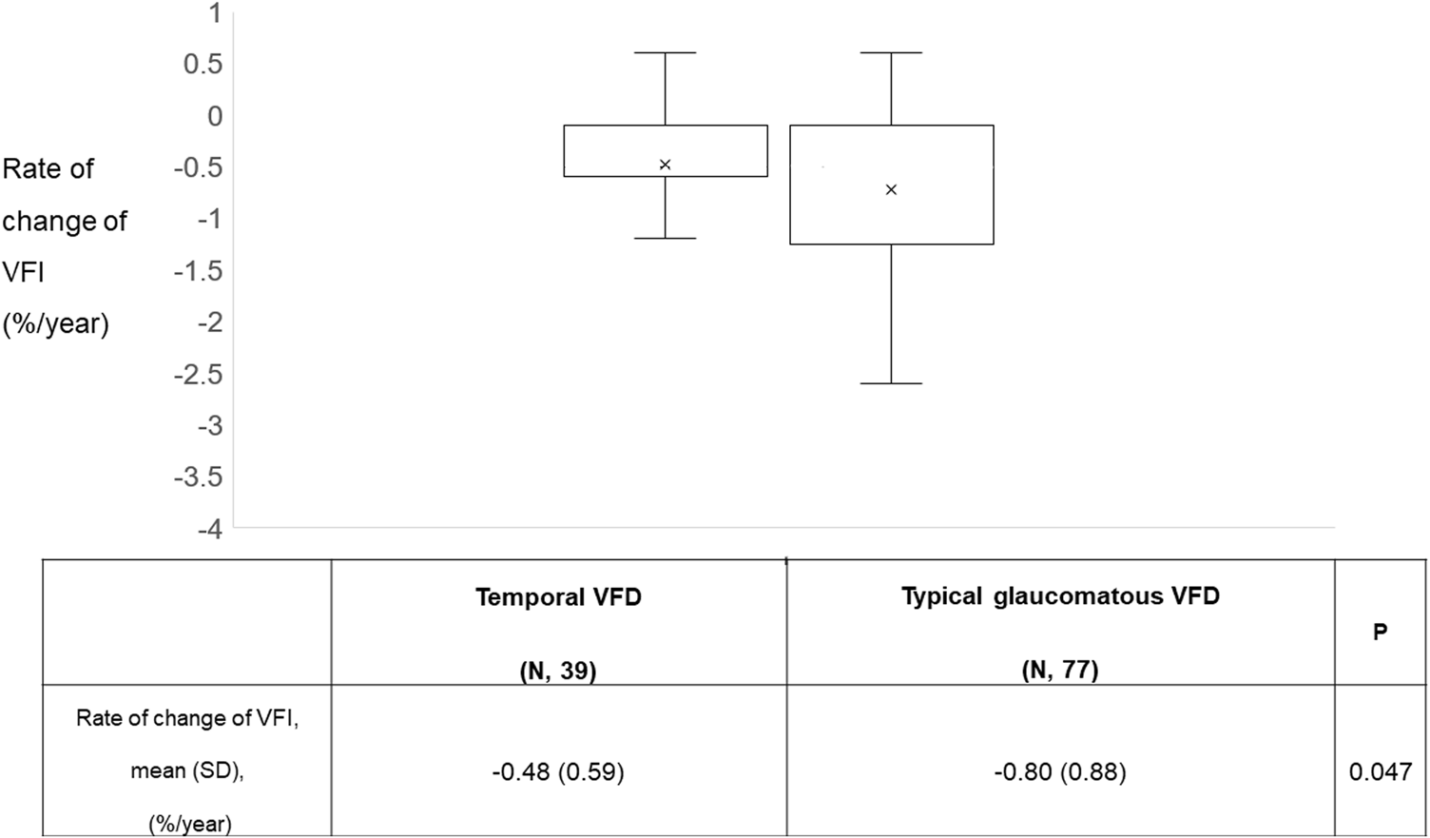
Glaucoma progression analysis using assessment with Trend- based analysis. Mean values of the rate of change of VFI (%/year) are shown. VFD indicates visual field defect; VFI, visual field index. Bold font indicates significant *P* values (*P* < 0.05). X in the box stands for the mean value for each group.

In terms of VFD progression, temporal VFD group had a noticeably slower progression rate than typical glaucomatous VFD group (− 0.49 ± 0.59, − 0.80 ± 0.88, *P* = 0.047) (Fig. 2).

In multivariate analysis to determine the parameters associated with VFD progression using trend-based GPA, manually measured cup to disc ratio, tilt ratio and change of β-zone PPA over years were noticeable factors in univariate analysis, however, in multivariate analysis, tilt ratio was of the most significant association (B, 1.063, *P* = 0.011) (Table 5).

**Table 5:** Linear regression analysis to Determine the Correlation between Variables and Visual Field defect progression (trend- based GPA) in All eyes (N, 116).

Variable		Univariate		Multivariate^1^	
		B	*P* value	B	*P* value
Age		− 0.009	0.214		
Baseline intraocular pressure		− 0.025	0.356		
Dependency on medication		0.082	0.798		
Central corneal thickness		− 0.003	0.196		
Axial length		0.012	0.741		
Manually measured cup to disc ratio		− 1.118	**0.015**		
Tilt ratio		1.063	**0.011**	1.063	**0.011**
Rotation degree		0.005	0.339		
Parameters for PPA	Initial PPA area	− 0.000001	0.613		
	Change of β-zone PPA over years	− 0.000043	0.184		
Optical coherence tomography	Average RNFL thickness	0.007	0.262		
	Average GCIPL thickness	0.008	0.447		
Visual field test	MD	− 0.057	0.145		
	PSD	− 0.009	0.810		
Type of visual field defect	Temporal	0.323	**0.074**		

*GPA* guided progression analysis, *PPA* peripapillary atrophy, *RNFL* retinal nerve fiber layer, *GCIPL* ganglion cell inner plexiform layer, *MD* mean deviation, *PSD* pattern standard deviation.

Average RNFL and GCIPL thicknesses were corrected with the Littmann’s formula.

Only variables with a *P* value < 0.10 in the univariate analysis were included in the multivariate model.

(Multivariate^1^ Analysis with stepwise or forward method, while Multivariate^2^ Analysis with backward method).

Bold font indicates significant *P* values (*P* < 0.05).

Meanwhile, in the analysis of the temporal VFD progression, manually measured cup to disc ratio, initial PPA area, change of β-zone PPA over years were significant in univariate analysis. Among those, it was change of β-zone PPA over years that had the most significance in multivariate analysis (B, − 0.000088, *P* = 0.003) (Table 6).

**Table 6:** Linear regression analysis to determine the correlation between variables and visual field (VF) defect progression (trend- based GPA) in temporal VF defect group (N, 39).

Variable		Univariate		Multivariate^1^	
		B	*P* value	B	*P* value
Age		− 0.009	0.277		
Baseline intraocular pressure		− 0.031	0.324		
Dependency on medication		0.361	0.155		
Central corneal thickness		− 0.002	0.342		
Axial length		− 0.018	0.699		
Manually measured cup to disc ratio		− 0.983	**0.043**		
Tilt ratio		0.697	0.115		
Rotation degree		0	0.960		
Parameters for PPA	Initial PPA area	− 0.000004	**0.059**		
	Change of β-zone PPA over years	− 0.000088	**0.003**	− 0.000088	**0.003**
Optical coherence tomography	Average RNFL thickness	0.009	0.123		
	Average GCIPL thickness	0.002	0.852		
Visual field test	MD	− 0.022	0.551		
	PSD	− 0.009	0.857		

*GPA* guided progression analysis, *MD* mean deviation, *PSD* pattern standard deviation, *RNFL* retinal nerve fiber layer, *GCIPL* ganglion cell inner plexiform layer, *PPA* peripapillary atrophy.

Average RNFL and GCIPL thicknesses were corrected with the Littmann’s formula.

Only variables with a *P* value < 0.10 in the univariate analysis were included in the multivariate model.

Bold font indicates significant *P* values (*P* < 0.05).

Based on the finding that increment in β-zone PPA was a significant factor in VFD progression in the temporal VFD group, we divided the patients of the temporal VFD group according to the presence of β-PPA progression. The patients with enlargement in β-zone PPA had lower tilt ratio, but vaster initial β-zone PPA, rim, and disc areas, and remarkable amount of β-zone PPA growth over years compared to those without enlargement in β-zone PPA (All *P*-values ≤ 0.002) (Table 7).

**Table 7:** Comparison of optic disc and optical coherence tomography parameters in eyes of the temporal visual field defect group in terms of presence of β-zone PPA progression.

Variable		Enlargement in β-zone PPA (N, 21)	No Enlargement in β-zone PPA (N, 14)	*P* value
Manually measured cup to disc ratio, mean (SD)		0.74 (0.19)	0.60 (0.20)	0.058
Tilt ratio, mean (SD)		1.35 (0.16)	1.61 (0.22)	** < 0.001**
Rotation angle, mean (SD), °		3.99 (21.38)	10.22 (10.95)	0.275
Area of Initial β-zone PPA, mean (SD), pixel		90,192.8 (50,250.9)	22,820.5 (20,504.0)	** < 0.001**
Change of β-zone PPA over years, mean (SD), pixel/year		5249.8 (3522.5)	479.4 (301.5)	** < 0.001**
Direction of β-zone PPA enlargement (No. (%)) (Inferotemporal/ Temporal/ Superonasal / Superotemporal)		10 / 7/ 3/ 1 (47.6%/ 33.3%/ 14.3%/ 4.8%)	NA	
Optic disc parameters	Rim area, mean (SD)	1.79 (0.86)	1.16 (0.50)	**0.018**
	Disc area, mean (SD)	3.46 (1.62)	2.05 (0.62)	**0.002**
	Average cup to disc ratio, mean (SD)	0.89 (0.21)	0.75 (0.25)	0.071
	Vertical cup to disc ratio, mean (SD)	0.82 (0.25)	0.68 (0.33)	0.162
	Cup volume, mean (SD)	0.32 (0.29)	0.27 (0.22)	0.633
Parapapillary RNFL thickness (μm)	Average, mean (SD)	90.49 (16.49)	85.79 (15.98)	0.415
	Superior, mean (SD)	99.92 (24.85)	86.55 (23.61)	0.125
	Nasal, mean (SD)	82.23 (23.86)	69.17 (23.83)	0.126
	Inferior, mean (SD)	89.63 (25.04)	105.45 (25.63)	0.082
	Temporal, mean (SD)	90.22 (22.06)	82.48 (24.34)	0.342
GCIPL thickness (μm)	Average, mean (SD)	80.22 (11.43)	77.35 (8.73)	0.462
	Minimum, mean (SD)	61.67 (24.09)	67.83 (16.15)	0.435
	Superior, mean (SD)	74.25 (18.40)	73.96 (12.49)	0.962
	Superonasal, mean (SD)	85.80 (17.15)	79.01 (18.80)	0.327
	Inferonasal, mean (SD)	82.15 (14.54)	79.57 (13.67)	0.633
	Inferior, mean (SD)	70.71 (16.23)	75.04 (8.51)	0.387
	Inferotemporal, mean (SD)	81.51 (19.84)	78.50 (9.16)	0.610
	Superotemporal, mean (SD)	86.96 (19.43)	77.56 (9.45)	0.116

Progression of β-zone PPA was defined with a change of β-zone PPA area over 1000 pixel /year.

*PPA* peripapillary atrophy, *NA* not available, *RNFL* retinal nerve fiber layer, *GCIPL* ganglion cell inner plexiform layer.

Values obtained from optical coherence tomography was corrected with the Littmann’s formula.

Student’s t test.

Bold font indicates significant *P* values (*P* < 0.05).

^a^Bonferroni correction for multiple measurement comparison was applied, and a level of significance was set at 0.002 (0.05/23).

Additionally, we further analyzed the data after applying 1:1 propensity-score matching for axial length, and a total of 54 eyes was included: 27 eyes for temporal VFD group and 27 eyes for typical glaucomatous VFD group. After 1:1 propensity-score matching, the mean axial length was 26.77 ± 1.15 mm for temporal VFD group, 26.21 ± 1.04 mm for typical glaucomatous VFD group (*P* = 0.061), and no significant differences except the percentage of the patient with eyedrops and the number of glaucoma medication were found (Supplementary Table S1 online). In terms of factors related to temporal VFD progression, unlike high myopic eyes, average RNFL thickness and manually measured cup to disc ratio were significantly associated with the VFD progression (B, 0.014, *P* = 0.022, B, − 1.086, *P* = 0.019, respectively) (Supplementary Table S2 online). However, in axial length -matched typical glaucomatous VFD group, there was no significant factors related to its VFD progression (Supplementary Table S3 online).

**Figure 3:**
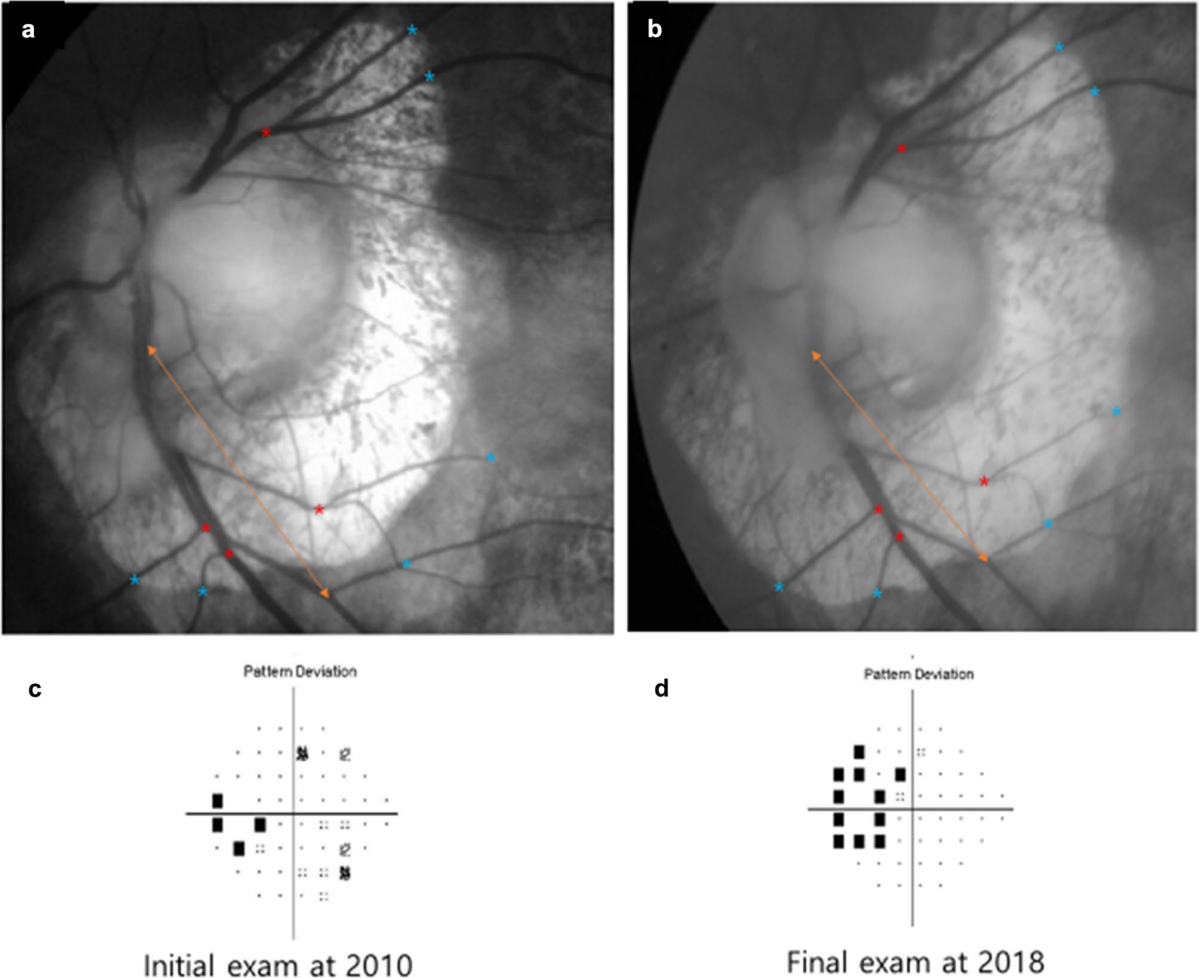
A Representative case with Enlargement of β-Peripapillary Atrophy. 59 years old female patient with axial length 28.41 mm showed that the overall change of β- PPA area over year was 12,115.8 pixel/year (**a** and **b**) and progression of the temporal visual field defect was observed (**c** and **d**).

A representative case of a patient with progressive temporal VFDs is shown in Fig. 3. Figure 3 highlights that the progression of temporal VFDs in eyes with β- PPA enlargement was prominent (Fig. 3a–d).

## Discussion

In this study, we found that eyes with myopia and temporal VFDs had prominent development and changes in β-PPA than typical glaucomatous VFD group. In the temporal VFD group, incremental β-PPA changes was associated with deterioration in the VF.

In this study, eyes with temporal VFDs showed myopic features including larger β-PPA areas than those with typical glaucomatous VFDs. The previous study suggested that myopic eyes with VFDs presented more extensive β-PPA than those without VFDs^11^, which were consistent with our findings. Regarding the presence of temporal VFDs in myopic eyes, there are some possible explanations. First, the β-PPA area is histologically known as having a complete loss of retinal pigment epithelial cells and decrease in retinal photoreceptors^12–15^. Temporal VFDs, including enlarged blind spots, might be connected to the histological changes in β-PPA. Secondly, we presume that RNFL axonal damage, as a consequence of myopic optic disc deformation, might affect the development of temporal VFDs. Along with axial elongation, defects like peripapillary scleral stretching and bending could be generated, and these defects might lead to damage to peripapillary deep structures including RNFL axons. According to Akagi et al^11^, in myopic patients with β-PPA, temporal scleral protrusion was detected in the eyes with VFD, and the angle of the temporal scleral bending correlated significantly with RNFL thickness above the scleral bending and VFD severity. In our study, significant differences in almost average RNFL thickness and average GCIPL thickness regions were found between the temporal VFD group and the typical glaucomatous VFD group. Although the values of the temporal VFD group were higher, they were far from within normal limit values. We presumed that this finding might reflect that RNFL damage also occurred concurrently with myopic optic nerve head deformation, but the degree of the damage might be subtle compared to general glaucomatous eyes.

In terms of the VFD progression rate, a significant difference was found between the two groups. Namely, although the progression rate was slower in temporal VFD group, there was a definite VF aggravation in some temporal VFD patients. This result was inconsistent with the previous study’s findings in young Chinese male patients with glaucoma or glaucoma suspect who had myopia and tilted optic discs and showed nonprogressive patterns of the VFD^16^. However, there were only 16 subjects in the study, which was not enough to generalize the findings. On the contrary to the precedent study, Ohno-Matsui et al^9^ reported that temporal VFDs were the most commonly observed VFD in high myopic, non-glaucomatous eyes, and those who had temporal VFDs showed aggravated VFDs.

As to the factor associated with VFD progression in all eyes, lower tilt ratio was the ultimate determinant. Tilt ratio as a contributor to VFD progression has been still on debate. Namely, Lee et al^17^ found that tilt ratio was not associated with VF progression rate, while Choy et al^18^ reported there were more VFDs found in eyes with increasing tilt ratio. In our study, compared to typical glaucomatous VFD group, temporal VFD group had greater tilt ratio and its progression rate was slower. We assumed that greater tilt ratio in the temporal VFD group might affect the results on VFD progression in multivariate analysis, even though the temporal VFD group itself did not remain as the significantly associated factor with progression of VFD.

Meanwhile, in myopic patients with the temporal VFD, our study revealed that incremental changes in β-PPA over time was associated with temporal VFD progression. According to previous findings, in glaucoma patients with myopia, growing β-PPA was associated with the progression of VFDs^19^. There also have been a report of changes in the extent of β-PPA in elderly myopic patients^20^. However, to our best knowledge, no study has found a direct relationship between changes in the β-PPA area and progression in VFDs in elderly myopic patients without definite glaucoma.

To explain these findings, we hypothesized some possible theories for the association between the progression of β-PPA and the aggravation of temporal VFDs. The first feasible explanation is that aggravation of temporal VFDs might be due to progressive degeneration in the outer retina, including the photoreceptor cells, followed by enlargement of the β-PPA area. Secondly, according to Chen et al.^21^, myopic eyes with profuse PPA showed choroidal thinning and decreased choroidal blood supply. In this regard, the disrupted choroidal blood circulation might result in insufficient blood supply to the ONH and thus, incremental changes in PPA could have an impact on the progression of VFDs. Lastly, the progression of temporal VFDs might be the co-result of retinal ganglion cell damage. This could be supported by our finding that the temporal VFD patients with progressive temporal VFD had thinner RNFL or GCIPL thickness as shown in supplemental Table 2.

In typical glaucomatous VF defect group, regarding factors related to VFD progression, a plethora of factors such as disc hemorrhage^22–25^, age^26^, baseline IOP^26^, inadequate IOP control^24,25,27^, tilt ratio^28–30^ and its direction^31,32^ were reported. However, there are still on-going controversies over these risk factors’ contribution to disease progression. In our study, none of the parameters were found to be significant. We presume these discrepancies between the studies might stem from assessment of patients with different severity; compared to the studies above, our study included the patients with the earliest stage of the disease in terms of MD. Also, small sample size might have led to this conclusion.

As for the direction of β-PPA enlargement, Bak et al.^33^ reported that inferotemporal area for primary open angle glaucoma (POAG), and the temporal area for normal eyes were the most frequent directions of expansion. Previous studies suggested that the direction of β-PPA enlargement was in accordance with that of RNFL defects in glaucomatous eyes^34,35^. Our study found that the direction of β-PPA enlargement was in the order of the inferotemporal area, followed by the temporal, superonasal, and superotemporal areas (Table 7). This distribution might imply that even myopic eyes without typical glaucomatous features could have some features of glaucomatous β-PPA.

## Limitations

First, our sample sizes were modest and this could mean other variables may not have been fully represented in the present study. However, the total follow-up period for all patients was more than six years, and this long-term follow-up might compensate for the weakness of the sample size. Second, due to weaknesses in a retrospective study including selection bias and lack of clarification of causal relationship, a prospective and longitudinal investigation is necessary. Third, since this study was conducted in a retrospective manner, we could not keep tracing changes of the AL. Nevertheless, we could not find the development or existence of posterior staphyloma, and thus, we presume there would have not been an extreme change in axial length. Lastly, thorough and specific analysis of other retinal layers including photoreceptor layer might have been helpful for better understanding of their impacts on temporal VF progression. Due to the fact that the whole OCT images were acquired from the Cirrus- OCT, it was impossible to perform more rigorous segmentation of the images. Future studies using swept-source OCT would be necessary to see the relationship between other retinal layers and the VFD progression.

## Conclusions

Myopic eyes with temporal VFDs demonstrated larger β-PPA areas, and slower VFD progression rate than glaucoma patients with typical VFDs. Among the myopic eyes with temporal VFDs, the eyes with increment in β-PPA area over years were predisposed to VFD progression. It has not been easy to tell high myopic patients with massive structural deformations around the ONH about the prognosis and predisposing factors associated with progression of atypical VFD. We believe our findings could be a tangible back-up resource when it comes to looking after those patients. It is never enough to stress the importance of extra-attention and care to myopic patients, particularly with presenting growing β-PPA.

### Patients and methods

This retrospective, observational study was approved by the Institutional Review Board (IRB) of Seoul St. Mary’s Hospital, Seoul, South Korea (KC20RASI0355) and followed the tenets of the Declaration of Helsinki. Written informed consent was waived due to the characteristics of the retrospective study design and the waiver was approved by the IRB of Seoul St. Mary’s Hospital, South Korea.

Patient data was collected from Feb 2010 to Feb 2020 from the electronic medical record and all patients were visiting the glaucoma clinic at Seoul St. Mary’s Hospital for glaucoma screening. A comprehensive ophthalmic assessment, including the measurement of best-corrected visual acuity, refraction, slit-lamp biomicroscopy, gonioscopy, Goldmann applanation tonometry, central corneal thickness using ultrasound pachymetry (Tomey Corporation, Nagoya, Japan), the estimation of axial length using ocular biometry (IOL Master; Carl Zeiss Meditec, Dublin, CA, USA), dilated stereoscopic examination of the optic disc and fundus, color disc photography, red-free RNFL photography (Canon, Tokyo, Japan), OCT (Cirrus OCT; Carl Zeiss Meditec), and Humphrey VF examination (24–2 Swedish Interactive Threshold Algorithm Standard program; Carl Zeiss Meditec) was carried out at initial work-up^36^. After the initial work-up, the patients were followed-up every 6 to 12 months according to their disease severity.

The inclusion criteria were: a best-corrected visual acuity of ≥ 20/40, a mean deviation (MD) better than − 6.00 decibels (dB) based on the Hodapp-Anderson-Parish criteria^37^, and five or more consistently reliable VFs (defined as a false-negative rate of < 15%, a false positive rate of < 15%, and fixation losses of < 20%). Patients were excluded based on any of the following criteria: less than five reliable VFs after excluding the initial one; a history of any retinal disease, including diabetic or hypertensive retinopathy or other retinal complications; a history of eye trauma or surgery, including glaucoma incisional surgery or laser procedure; another optic nerve disease besides glaucoma; or a history of systemic or neurological diseases that might affect the VF. Additionally, to exclude eyes with pathologic myopia, patients who showed either posterior staphyloma or myopic maculopathy on wide photography (Optos, Dunfermline, UK) or OCT images from Cirrus—OCT macular scan were refrained from participating in the study.

If both eyes were eligible, one eye was randomly selected from each patient that met the inclusion and exclusion criteria.

### Definition of the temporal VFD group

**Figure 4:**
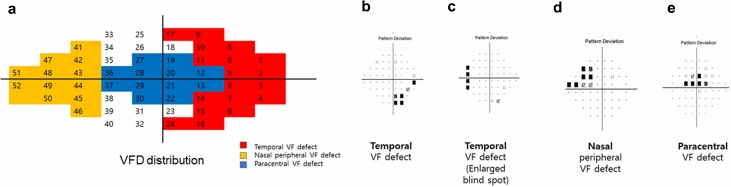
Visual Field Defect (VFD) regions. The distribution of the VFD regions is shown on (**a**). Blue region stands for paracentral VFD, while red region is classified as temporal VFD. Rest regions fall into the category for peripheral VFD. X regions corresponds to the blind spots. (**b**) and (**c**) show examples of the temporal VFD, while (**d**) and (**e**) describe nasal peripheral VFD, and paracentral VFD, respectively.

To be included in the temporal VFD group, the patients were required to meet the following criteria: temporal VFDs defined by VFDs that were restricted to either the temporal edges of the field (Fig. 4b) or around the blind spot (Fig. 4c) and myopic eyes with an axial length ≥ 24.5 mm without definite glaucoma diagnosed by the criteria described below.

### Definition of the typical glaucomatous VFD groups: Peripheral nasal step or paracentral scotoma groups

The peripheral nasal and paracentral scotoma groups were recruited from patients with early open-angle glaucoma with MD better than − 6.00 decibels (dB). Peripheral nasal VFD was defined as a glaucomatous VFD in one hemifield in the nasal periphery outside 10 degrees of fixation with no involvement within the central 10 degrees and the presence of at least three contiguous test points within the same hemifield on the pattern standard deviation (PSD) plot at *P* < 1%, with at least one at *P* < 0.5% (Fig. 4d)^38^. The paracentral VFD group included patients with a glaucomatous VFD in one hemifield within 10 degrees of fixation, with at least one point at *P* < 0.5% lying at the two innermost paracentral points and no VFDs outside the central 10 degrees (Fig. 4e)^38^. For those with two VFDs, points on the edge of the VF field or those directly around the blind spot were excluded. To elucidate specific features of myopic eyes with temporal VFD, we classed patients into two groups: 1) temporal VFD group and 2) typical glaucomatous VFD group, a combination of patients with peripheral nasal VFD and paracentral VFD. Figure 4 details specific distribution of the VFD types.

For glaucoma diagnosis, following criteria were applied to patients: glaucomatous optic disc appearances (presenting diffuse or localized rim thinning, a notch in the rim, or a cup-to-disc ratio higher than that of the other eye by > 0.2), VF changes consistent with glaucomatous VFD such as a cluster of ≥ 3 non-edge points on the pattern deviation plot with a probability of < 5% of the normal population, with one of these points having a probability of < 1%, a pattern standard deviation with *P* < 5%, or a Glaucoma Hemifield Test result outside the normal limits in a consistent pattern on two qualifying VFs—these results were confirmed by two glaucoma specialists (K.I.J. and C.K.P.)-, and an open-angle evident on gonioscopy.

### Assessment of VFD progression

Defects VFD progression was evaluated by the glaucoma progression analysis (GPA) II trend analysis algorithm of the visual field index (VFI)^39^. The VFI allocates more importance to central points than to peripheral points and is adjusted for age. This parameter is expressed in percent, with 100% a perimetrically normal visual field, and 0% a totally blind one. To minimize the effect of cataracts, a pattern deviation probability map was used to identify the test points with normal sensitivity (100% VFI); those showing a relative loss, which is scored as a function of total deviation and an age-corrected normal threshold; and those with no sensitivity (0% VFI). If the change slope of the VFI was statistically significant (*P* < 0.05) by GPA, a noticeable trend was considered.

### Assessment with optical coherence tomography

OCT images were obtained by scanning the eyes with the Cirrus OCT using software version 6.0. Once the ONH was centered, the laser scanned a 6 × 6 mm area, which captured a cube of data consisting of 200 A scans from 200 linear B scans (40,000 points) over approximately 1.5 s (27,000 A scans). After determining the optic disc, the OCT algorithms automatically placed a circle of 3.46 mm in diameter around the disc. Software version 6.0 provided the data of the ONH parameters, including the disc area, the rim area, the cup-to-disc ratio, the vertical cup-to-disc ratio, and the cup volume. The RNLF thickness within a 3.46 mm diameter circle (256 A scan) automatically positioned around the ONH was measured and analyzed in 17 parameters: average, superior, nasal, inferior, temporal, and 12 clock-hour sectors. Regarding the GCIPL thickness, the GCIPL thickness within a 6 × 6 × 2 mm (14.13mm^2^) elliptical annulus around the fovea was measured and computed by the GCA algorithm embedded in the OCT. The annulus cube was 1 mm in inner vertical diameter, 4 mm in outer vertical diameter, 1.2 mm in inner horizontal diameter, and 4.8 mm in outer horizontal diameter, excluding the central portions of the fovea where the layers are thin and difficult to defect. GCIPL thickness was then analyzed in eight parameters: average, minimum, superior, superonasal, inferonasal, inferior, inferotemporal, and superotemporal^40,41^.

To correct axial length -related ocular magnification, the Littmann’s formaula (t = p*q*s) was applied^42,43^, and additionally, the ONH area was modified with the modified formula (t^2^ = p^2^*q^2^*s^2^) as well^44,45^.

Poor images due to involuntary saccade, misalignment, or artifacts, and signal strengths < 6 were excluded.

### Measurement of Optic Disc Tilt, Torsion, and PPA

Digital retinal photographs centered on the optic disc and macular were used for assessing the degree of optic disc tilt, torsion, and PPA. They were measured by a glaucoma specialist (J. L) using the National Institutes of Health image analysis software (ImageJ version 1.52; available at http://rsb.info.nih.gov/ij/index.html; developed by Wayne Rasband, National Institutes of Health, Bethesda, MD, USA).

**Figure 5:**
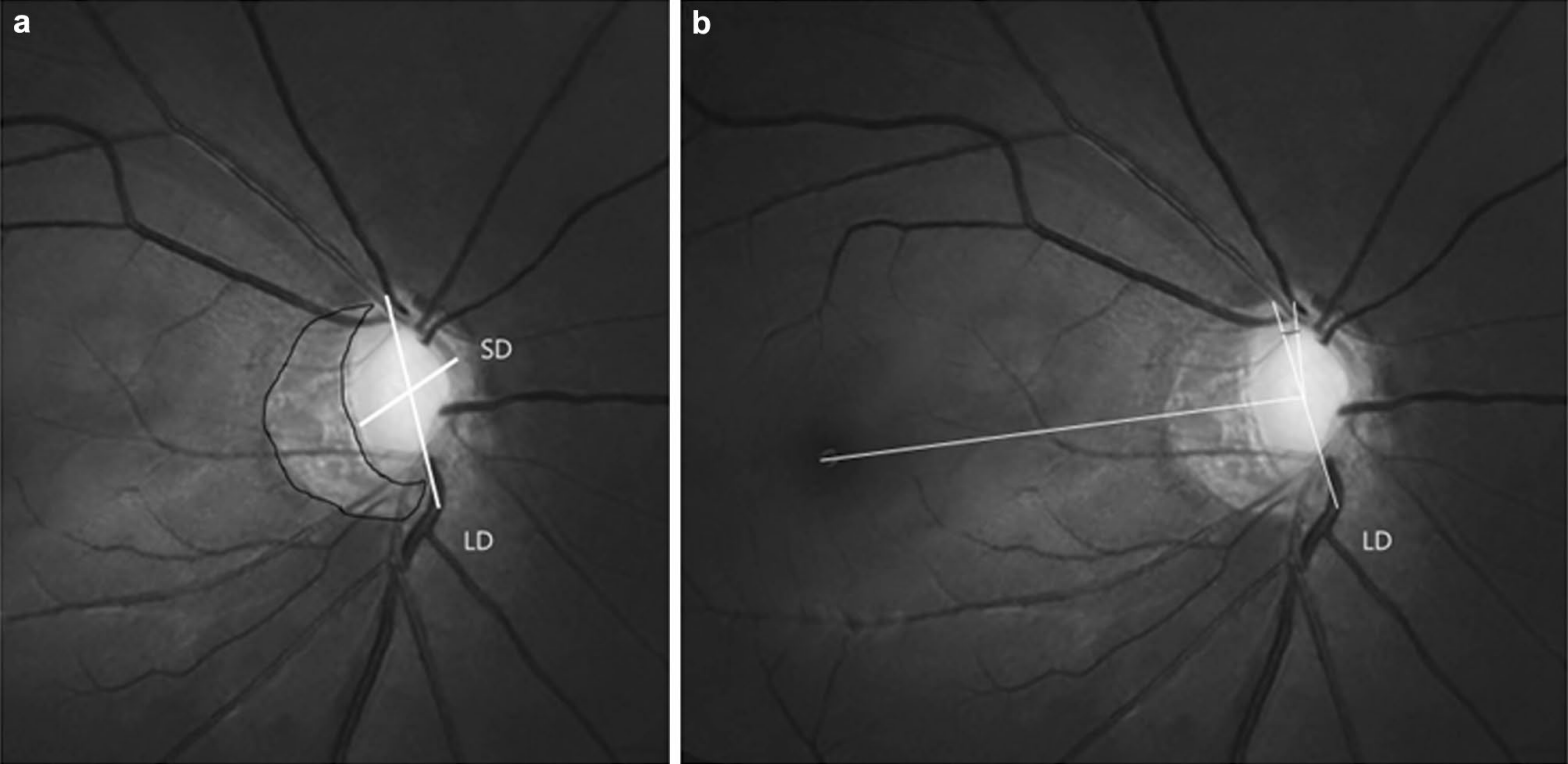
Identification of Tilt ratio, β-Peripapillary Atrophy area, and Torsion degree. (**a**) Tilt ratio was defined as the ratio between the longest diameter and the shortest diameter of the optic disc. The border of β-peripapillary atrophy area was drawn manually with ImageJ software, and the pixel are was calculated automatically. (**b**) Torsion degree was defined as the angle between the longest diameter and the vertical meridian of the optic disc, which meant a vertical line 90 degrees from the reference line connecting the fovea to the center of the optic disc. *LD* longest diameter, *SD* shortest diameter.

With reference to the precedent study^46^, optic disc tilt was defined as the ratio between the longest and shortest diameters of the optic disc was more than 1.30 (Fig. 5a). Optic disc torsion was measured by the deviation of the long axis of the optic disc from the vertical meridian and was defined when the degree of the deviation was more than 15° (Fig. 5b). The β-PPA, an inner crescent of chorioretinal atrophy with visible sclera and choroidal vessels, was drawn with a mouse to trace the disc and the PPA margins and the area of β-PPA was calculated using the ImageJ software (Fig. 5a). Photographic magnification was corrected with Littmann’s method^47,48^. The area of βPPA was measured by two authors who were masked to the clinical data (J.L and K.I.J). The values were determined as the mean of the measurements performed by the two authors. A third adjudicator (C.K.P) resolved disagreements between two observers.

### Subgroup analysis

To determine whether there were different clinical characteristics or risk factors related to the presence of progression of β- zone PPA, we performed subgroup analyses in the temporal VFD group. Specifically, the temporal VFD group was divided into two groups, one with true β-PPA enlargement and the other without true β-PPA enlargement. To assess β-PPA enlargement, the initial and final areas of β-PPA were measured and the change between areas was divided by the total follow-up year. True β-PPA enlargement was defined as increments of over 1000 pixels per year.

Furthermore, we analyzed the data after applying 1:1 propensity-score matching for axial length because there is a significant difference in axial length between groups.

### Statistical analysis

All statistical analyses were performed using the SPSS statistical package (SPSS, Inc, Chicago, IL, USA). A propensity score analysis was performed to match eyes between the temporal VFD group and the typical glaucomatous VFD group regarding axial length. By using multiple logistic regression analysis, the propensity scores were calculated^49^. Using predicted probabilities, we sought to match a patient in the temporal VFD group with the closest patient in the typical glaucomatous VFD group using propensity score values. Applying the Greedy 5 → 1 digit match algorithm^50^, we created propensity score-matched pairs without replacement (a 1:1 match). Unless this could be achieved, the algorithm proceeded sequentially to the next highest digit match (4-,3-,2-, or 1- digit match) until no further matches were possible.

Test–retest variability was estimated adopting the intraclass correlation coefficient (ICC). ICC scores ≥ 0.75, 0.40–0.75, and ≤ 0.40 are considered to be excellent, moderate, and poor, respectively^51^. Student’s t-test was adopted to compare the differences between the groups. The chi-square test was applied to compare frequencies. A *P-*value of less than 0.05 was considered statistically significant. Linear regression analysis was used to determine the factors associated with VFD progression in the total group and the temporal VFD group. The variables with significance at *P* < 0.10 in univariate analysis were included in the multivariate model. *P* < 0.05 was considered to represent statistical significance. In order to adjust multiple comparisons, Bonferroni correction was used when needed.

## Supplementary Information

Supplementary Information 1.

Supplementary Information 2.

Supplementary Information 3.
