# Supplementary material for: Characteristics of progressive temporal visual field defects in patients with myopia
Source: Sci Rep. 2021 Apr 30;11:9385. doi: 10.1038/s41598-021-88832-1 (PMC8087792; doi:10.1038/s41598-021-88832-1)
Supplement: Supplementary file 1 — Supplementary Information 1. [file 41598_2021_88832_MOESM1_ESM.docx]

**Supplement Table 1 Patients demographics after 1:1 Propensity score match (Total 54 eyes)**

|  | **Temporal VFD**  (N, 27) | **Typical glaucomatous VFD**  (N, 27) | P value |
| --- | --- | --- | --- |
| Age, mean (SD), year | 48.74 (13.42) | 48.52 (10.37) | 0.946 ^a^ |
| Female, No. (%) | 20 (74.1) | 13 (48.1) | 0.051 ^b^ |
| Laterality, No. (%) |  |  |  |
| OD | 15 (55.6) | 16 (59.3) | 0.783 ^b^ |
| OS | 12 (44.4) | 11 (40.7) |  |
| HTN (No. (%)) | 0 (0) | 2 (7.4) | 0.150 ^b^ |
| DM (No. (%)) | 0 (0) | 1 (3.7) | 0.313 ^b^ |
| Aspirin (No. (%)) | 0 (0) | 2 (7.4) | 0.150 ^b^ |
| Migraine (No. (%)) | 3 (11.1) | 3 (11.1) | 1.000 ^b^ |
| Cold hands/ feet (No. (%)) | 2 (7.4) | 3 (11.1) | 0.639 ^b^ |
| Baseline IOP, mean (SD), mmHg | 15.85 (3.67) | 16.04 (3.17) | 0.843 ^a^ |
| Final IOP, mean (SD), mmHg | 14.89 (3.11) | 14.44 (3.29) | 0.612 ^a^ |
| Number of patients with medication (No. (%)) | 20 (74.1) | 37 (100) | 0.013^b^ |
| Number of medications, mean (SD), n | 1.11 (0.85) | 1.85 (0.78) | 0.002 ^a^ |
| CCT, mean (SD), ㎛ | 545.35 (53.37) | 543.96 (43.46) | 0.918 ^a^ |
| Axial length, mean (SD), mm | 26.77 (1.15) | 26.21 (1.04) | 0.068 ^a^ |
| Number of VF test, mean (SD), n | 6.68 (1.38) | 6.11 (1.10) | 0.052 ^a^ |
| Total follow-up period, mean (SD), month | 86.43 (14.49) | 80.90 (20.20) | 0.061 ^a^ |
| MD, mean (SD), dB | -2.39 (2.46) | -3.11 (1.44) | 0.198 ^a^ |
| PSD, mean (SD), dB | 4.98 (2.28) | 4.85 (1.92) | 0.810 ^a^ |

Abbreviations: VFD= visual field defect; HTN= hypertension; DM= diabetes mellitus; IOP= intraocular pressure; n= number; CCT= central corneal thickness; VF= visual field; MD= mean deviation; PSD= pattern standard deviation; dB=decibel.

Mean values are presented with standard deviations ^a^ Student’s t-test after Propensity score match, ^b^ chi-squared test.

Bold font indicates significant p values (p < 0.05).
