# Supplementary material for: Characteristics of progressive temporal visual field defects in patients with myopia
Source: Sci Rep. 2021 Apr 30;11:9385. doi: 10.1038/s41598-021-88832-1 (PMC8087792; doi:10.1038/s41598-021-88832-1)
Supplement: Supplementary file 2 — Supplementary Information 2. [file 41598_2021_88832_MOESM2_ESM.docx]

**Supplement Table 2. Linear regression analysis to determine the correlation between variables and visual field (VF) defect progression (trend- based GPA) in temporal VF defect group (N, 27)**

| **Variable** | | **Univariate** | | **Multivariate^1^** | |
| --- | --- | --- | --- | --- | --- |
|  |  | **B** | **P value** | **B** | **P value** |
| Age | | 0.001 | 0.943 |  |  |
| Baseline intraocular pressure | | -0.011 | 0.685 |  |  |
| Dependency on medication | | -0.097 | 0.702 |  |  |
| Central corneal thickness | | 0 | 0.908 |  |  |
| Axial length | | 0.095 | 0.308 |  |  |
| Manually measured cup to disc ratio | | -0.864 | **0.070** | -1.086 | **0.019** |
| Tilt ratio | | 0.526 | 0.160 |  |  |
| Rotation degree | | 0.004 | 0.474 |  |  |
| Parameters for PPA | Initial PPA area | -0.000002 | 0.332 |  |  |
|  | Change of β-zone PPA over years | -0.000061 | **0.075** |  |  |
| Optical coherence tomography | Average RNFL thickness | 0.011 | **0.094** | 0.014 | **0.022** |
|  | Average GCIPL thickness | 0.014 | 0.173 |  |  |
| Visual field test | MD | -0.077 | 0.340 |  |  |
|  | PSD | -0.026 | 0.568 |  |  |

Abbreviations: GPA= guided progression analysis; PPA= peripapillary atrophy; RNFL= retinal nerve fiber layer; GCIPL=ganglion cell inner plexiform layer; MD= mean deviation; PSD= pattern standard deviation.

Average RNFL and GCIPL thicknesses were corrected with the Littmann’s formula.

Only variables with a P value <0.10 in the univariate analysis were included in the multivariate model

Bold font indicates significant p values (p < 0.05).
