# Supplementary material for: Characteristics of progressive temporal visual field defects in patients with myopia
Source: Sci Rep. 2021 Apr 30;11:9385. doi: 10.1038/s41598-021-88832-1 (PMC8087792; doi:10.1038/s41598-021-88832-1)
Supplement: Supplementary file 3 — Supplementary Information 3. [file 41598_2021_88832_MOESM3_ESM.docx]

**Supplement Table 3. Linear regression analysis to determine the correlation between variables and visual field (VF) defect progression (trend- based GPA) in typical glaucomatous VF defect group (N, 27)**

| **Variable** | | **Univariate** | |
| --- | --- | --- | --- |
|  |  | **B** | **P value** |
| Age | | -0.031 | 0.270 |
| Baseline intraocular pressure | | -0.015 | 0.892 |
| Dependency on medication | | 0.332 | 0.442 |
| Central corneal thickness | | -0.007 | 0.340 |
| Axial length | | 0.062 | 0.819 |
| Manually measured cup to disc ratio | | -2.326 | 0.164 |
| Tilt ratio | | 2.397 | 0.236 |
| Rotation degree | | 0.016 | 0.330 |
| Parameters for PPA | Initial PPA area | -0.000004 | 0.937 |
|  | Change of β-zone PPA over years | 0 | 0.423 |
| Optical coherence tomography | Average RNFL thickness | 0.018 | 0.626 |
|  | Average GCIPL thickness | 0.064 | 0.184 |
| Visual field test | MD | -0.027 | 0.885 |
|  | PSD | -0.104 | 0.462 |

Abbreviations: GPA= guided progression analysis; PPA= peripapillary atrophy; RNFL= retinal nerve fiber layer; GCIPL=ganglion cell inner plexiform layer; MD= mean deviation; PSD= pattern standard deviation.

Average RNFL and GCIPL thicknesses were corrected with the Littmann’s formula.

Only variables with a P value <0.10 in the univariate analysis were included in the multivariate model

Bold font indicates significant p values (p < 0.05).
